# Role of Nano-silver and the Bacterial Strain *Enterobacter cloacae* in Increasing Vase Life of Cut Carnation ‘Omea’

**DOI:** 10.3389/fpls.2017.01590

**Published:** 2017-09-13

**Authors:** Aung H. Naing, Nay M. Win, Jeung-Sul Han, Ki B. Lim, Chang K. Kim

**Affiliations:** Department of Horticultural Science, Kyungpook National University Daegu, South Korea

**Keywords:** antioxidant activity, bacterial blockage, ethylene production, relative fresh weight, relative gene expression, nano-silver, *Enterobacter cloacae*, carnation

## Abstract

We investigated the role of nano-silver (NAg) and the bacterial strain *Enterobacter cloacae* in increasing the vase life of cut carnation flowers ‘Omea.’ NAg treatment extended vase life of the flowers by increasing relative fresh weight, antioxidant activities, and expression level of the cysteine proteinase inhibitor gene (*DcCPi*), and by suppressing bacterial blockage in stem segments, ethylene production and expression of ethylene biosynthesis genes and *DcCP1* gene, compared with the control. Out of all the treatments, administration of 25 mg L^-1^ NAg gave the best results for all the analyzed parameters. Interestingly, application of *E. cloacae* also extended the vase life of cut flowers by 3 days in comparison with control flowers, and overall, showed better results than the control for all the analyzed parameters. Taken together, these results demonstrate the positive role of NAg and *E. cloacae* in increasing the longevity of cut carnation flowers, and indicate that this effect is brought about through multiple modes of action.

## Introduction

Carnation is one of the most economically important cut flowers and plays a significant role in the florist trade (Ali et al., 2008). However, post-harvest senescence occurs within a few days, and is a major limitation in the marketing of cut carnation flowers. Generally, early post-harvest senescence is caused by the production of ethylene in petals via transcriptional regulation of ACS gene and ACC oxidase (ACO) gene involved in ethylene synthesis, which causes petal in-rolling, and by the accumulation of bacteria on cut-stem surfaces, which produces extracellular polysaccharides (EPSs) that block xylem vessels and thereby increase hydraulic resistance, subsequently leading to a reduction in water uptake by the stem and premature wilting (van Doorn et al., 1995; Bowyer et al., 2003). In order to increase vase life of cut flowers, efforts have been made to reduce ethylene production in petals using various ethylene inhibitors, such as STS, nitric oxide (NO), and 1-MCP (Bowyer et al., 2003; Ichimura and Niki, 2014; In et al., 2015), which suppress the transcript levels of ethylene biosynthesis genes (ACS and ACO) involved in ethylene synthesis. Similarly, the bacteria-induced xylem blockage, which causes reduction in water uptake, can be prevented using various chemical compounds containing silver ions (Ag^+^) that has antimicrobial activity to inhibit the growth of bacteria and other microorganisms (Feng et al., 2000; Rai et al., 2009).

Recently, many researchers have demonstrated the role of NAg in increasing the vase life of various cut flowers, such as gerbera, gladiolus, and rose (Solgi et al., 2009; Rafi and Ramezanian, 2013; Hassan et al., 2014; Li et al., 2017), in which, NAg could suppress not only ethylene biosynthesis genes but also bacterial growth in the cut stems, which are mainly associated with longevity of cut flowers. However, they did not investigate the effect of NAg on ethylene production, antioxidant activities, and transcriptional regulation of genes related to ethylene and petal senescence, all of which determine the duration of vase life of cut flowers. Notably, none of the previous reports has described the role of NAg in increasing vase life of cut carnation flowers via suppression of ethylene production, inhibition of bacterial growth, and maintenance of antioxidant activities. Because of insufficient data, the role of NAg in extending vase life of cut flowers, especially carnation, is not well-understood.

Although accumulation of bacteria in cut-stem surface or xylem vessel shortened vase life of cut flowers (Solgi et al., 2009; Hassan et al., 2014; Li et al., 2017), some studies claimed that shortening of vase life by bacteria depended on the type of bacteria present therein (van Doorn et al., 1991; Jacob and Kim, 2010; Carlson et al., 2015). In addition, recently, Carlson et al. (2015) also reported that two bacterial strains (*Pseudomonas fulva* and *Escherichia coli*), which are also known as biocontrol bacteria, enhanced vase life of cut *Zinnia elegans*. In this study, the biocontrol bacteria *Enterobacter cloacae* was mostly observed in the stem segments, thus, we are interested to investigate on how it is involved in vase life of the carnation.

In this study, we investigated the role of NAg in the process of senescence of cut flowers by determining RFW of cut flowers of carnation and other flower senescence associated parameters, such as blockage of xylem vessels, ethylene production, expression of ethylene biosynthesis-related genes, and variation in antioxidant activity. In addition, role of biocontrol bacteria *E. cloacae* in vase life of cut flowers was also investigated.

## Materials and Methods

### Plant Material

Cut flowers of carnation ‘Omea,’ graded as marketable quality, were harvested from a greenhouse during March and April of 2016, in Youngju region, located 130 km away from the laboratory, and used in this experiment. The greenhouse is set with the favorable environmental factors, such as temperature (at 22–24°C during the day and 16°C during night), light (14 h photoperiod with the help of supplemental lighting, approximately 45.36 molm^-2^d^-1^), and relative humidity (70%), for production of quality cut flowers. Therefore, preharvest conditions that negatively affect the keeping quality of cut flowers (e.g., 24 h photoperiod and relative humidity above 85%) were avoided (Fanourakis et al., 2013, 2015; van Meeteren and Aliniaeifard, 2016), In the laboratory, the cut flower stems were placed in distilled water and re-cut to achieve a length of about 40 cm, in accordance with the commercial practice. In addition, leaves that were in direct contact with the vase water were carefully removed by hand.

### Treatment with Nano-silver Nanoparticles (NAg)

In a preliminary experiment, vase life of carnation ‘Omea’ varied with different concentrations of NAg (1–50 mg L^-1^; Sigma) used (data not shown), out of which two concentrations, i.e., 25 and 50 mg L^-1^ NAg, were found to be optimal for long vase life. Thus, in this study, we chose these two concentrations to reconfirm their positive effect on vase life of the cultivar. Briefly, 15 cut stems having approximately equivalent fresh weight were placed in plastic bottles (1.5 L volume) containing NAg (0, 25, and 50 mg L^-1^) dissolved in 500 mL of distilled water for 24 h. Next, the treated stems were thoroughly washed under tap water and replaced in the vases containing 500 mL distilled water. The vases were then maintained in a growth chamber at light intensity 20 μmol^-2^ s^-1^, temperature 23°C, and relative humidity 60–70% for 12 h. Each treatment was applied to three vases and each experiment was conducted in triplicates.

While selecting cut stem quality, in order to be free of bias, the initial fresh weight of all flower stems in each vase was recorded, after which five flowers from each vase were selected to evaluate RFW and vase life throughout the experiment. The remaining 10 flowers in each vase were used for isolation of bacteria, investigation of xylem vessel blockage, estimation of ethylene production, transcriptional analysis of ethylene biosynthesis genes and petal senescence-related genes, and measurement of antioxidant activities.

### Isolation, Quantification, and Identification of Bacteria

On the sixth day of vase period, when control flowers (non-NAg treated flowers) started to show petal senescence or wilting, about 1-cm long stem segments from the proximal ends of both control and Nag-treated flower stems were excised using sterile scalpel blades and the bacterial densities inside these stem segments were analyzed. Briefly, the segments were surface-sterilized with 70% ethanol and further excised into small (∼10 mm) pieces. The pieces were then transferred into 2-mL sterile tubes containing 1 mL sterile normal saline (0.9%). Bacteria were dislodged by vortexing for 5 min. An aliquot of bacterial suspension (0.1 mL) was then serially diluted with sterile 0.9% normal saline, and the diluted suspensions (0.1 mL) were spread over LB media and incubated at 30°C for 36 h. Each treatment was applied to three stems, and each stem was incubated on three plates.

After 36 h of incubation, number of bacteria (colony forming units per milliliter or cfu mL^-1^) was counted and compared for all the treatments. Many phenotypic differences were observed in the bacteria obtained from control stem segments, in terms of color and size of the bacterial colonies. To identify the different types of bacteria, we picked up single colonies of each type of bacteria and recultured them on LB agar medium. Next, the pure single colonies obtained were sent to a sequencing company (Microgent) for 16S rDNA-based PCR analysis.

### Scanning Electron Microscopy (SEM) Observation

Bacterial growth in the xylem vessels was investigated using scanning electron microscopy (SEM) (JEOL, Ltd, Tokyo, Japan). Stem segments (3 mm long) were excised from the base of each stem (control and NAg treated stems), using scalpel blades on the sixth day of vase period. The stem segment samples were immediately fixed in formalin-acetic acid-alcohol (FAA) and kept overnight, following the protocol of Naing et al. (2015). Subsequently, the samples were dehydrated for 10 min using serial concentrations of ethanol (25, 50, 70, 85, and 100%). Then, the dehydrated samples were dried until the critical point at room temperature. Next, the samples were coated with gold-palladium on a Quick Cool Coater (Sanyu-Denshi, Japan), examined under an SEM (JEOL, Ltd), and photographed.

### Effect of *Enterobacter cloacae* on Vase Life of the Carnation ‘Omea’

Bacteria growing in the stem segments were identified, and out of all these bacterial types, the most abundant bacteria (40%) was observed to be *E. cloacae*. This bacterial strain is a biocontrol agent capable of killing other microorganisms present in the vase solution. Thus, we investigated the effect of this bacterial strain on vase life of the carnation ‘Omea.’ As in the NAg treatment, cut stems having approximately equivalent fresh weight were placed in vases containing 500 mL distilled water (control) or 10^7^ cfu mL^-1^ of the bacterial solution (treatment). The vases were maintained in the same growth chambers under the same conditions that were used in the above experiment. In addition, physiological and molecular analyses were performed as in the previous experiment. There were three vases per cultivar and each experiment was conducted in triplicates.

### Vase Life and RFW

Post-harvest life of each flower was determined when it reached the stage where more than one-third of its petals showed in-rolling, browning, or loss of ornamental value. The fresh weight of each cut stem was measured after intervals of 3 days, and the RFW was calculated using the formula: RFW (%) = (FWt/FW0) × 100; where FWt is the fresh weight of the stem (g) on days 3, 6, or 9, and FW0 is the initial fresh weight of stem (g) on day 1 (He et al., 2006). In each experiment, five flowers were used per treatment with three replications.

### Measurement of Ethylene

For measurement of ethylene, 5 g of petals from each treatment were weighed and sampled after different time intervals (Day 3, Day 6, and Day 9). They were placed in a 50 mL glass tube and enclosed with a rubber septum for 16 h at 20°C. An aliquot of the accumulated gas (1 mL) was withdrawn using a 1 mL syringe that was allowed to penetrate through the septum. Subsequently, this gas was analyzed to detect the presence of ethylene using a gas chromatograph (GC-2010, Shimadzu). In all the experiments, three syringes (three replicates) were used for each treatment.

### RNA Extraction and Quantitative Real-time PCR (qRT-PCR) Analysis

Total RNA was extracted from 100 mg of petals using the RNeasy Plant Mini Kit (Qiagen, Hilden, Germany). cDNA was synthesized from 1 μg of the total RNA using an oligo dT_20_ primer, with the help of a reverse transcription kit (ReverTra Ace-aì, Toyobo, Japan). Transcript levels of 1-aminocyclopropane-1-carboxylate oxidase (*DcACO1*) and 1-aminocyclopropane-1-carboxylate synthase (*DcACS1*), and petal senescence-related genes (cysteine proteinase gene *DcCP1* and its inhibitor gene *DcCPi*) were measured using a StepOnePlus Real-Time PCR system (Thermo Fisher Scientific, Waltham, MA, United States) (Ai et al., 2016). To confirm the amount of template RNA, a fragment of the carnation actin (*DcACT*) gene was used as internal control. The primers and PCR conditions used for detecting the expression levels of these genes are listed in **Table 1**. In all the experiments, five samples per treatment were used, and each analysis was repeated three times.

**Table 1:** Primer sequences used for detecting genes related to ethylene production and petal senescence by qRT-PCR.

Gene	Primer sequence (5′-3′)	PCR condition
*ACO1*	F- CCG AGC AAC TGT TGG ACT TG	
	R- AGA GAA TGA TGC CAC CAG CG	
*ACS1*	F- TCC AGG GTT TAG GGT TGG GA	
	R- CCT TCC TAC AAA CGC CTC GT	
		95°C (10 min) → [95°C (15 s) →57°C (1 min) →72°C (35 s)] ×40 cycles → 95°C (15 s) →59.3°C (1 min) → 95°C (15 s)
*CPi*	F- GGT GAA ACC GTG GGT GAA CT	
	R- CCT TCC AGA AAC ATG CTC CG	
*CP1*	F- TCA TCA TGC CCT AGT GCG AC	
	R- TGT TGG GTG TTA CAG ACG GG	
*Actin*	F- GCA CGG TAT CGT CAC CAA CT	
	R- AGC CTT TGG GTT AAG AGG CG	


### Determination of Antioxidant Activities

Petals were collected from the flowers on day 6 after treatment, when ornamental value of most of the control flowers had faded, and were frozen for analysis of antioxidant activities.

To examine DPPH and ABTS activities, 5 g of frozen petals were used and the analyses were performed following the method of Kim et al. (2014). For analysis of the total polyphenol and total flavonoid contents, we followed the method of Dewanto et al. (2002). Five samples were used per treatment, and each analysis was repeated three times.

### Statistical Analysis

For each experiment, data are presented as means ± standard errors (SE) of three replicates.

## Results

### Vase Life and RFW

**FIGURE 1:**
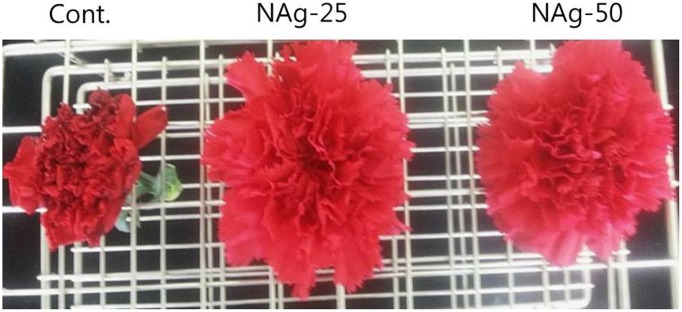
Effect of nano-silver (NAg) that delayed petal senescence in flowers of the carnation ‘Omea.’ The photograph was taken on day 6 after treatment.

**FIGURE 2:**
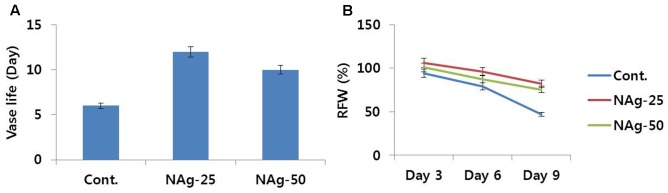
Effect of NAg on vase life **(A)** and RFW **(B)** of flowers of cut carnation ‘Omea’ during the vase period. Data represents the means ± SE of three replicates.

All flowers treated with NAg showed efficient water uptake as compared with control flowers and survived without showing any symptom of senescence until day 6, whereas control flowers exhibited petal senescence by day 6 (**Figure 1**). On day 7, rapid petal senescence was observed in all the control flowers, whereas such senescence symptoms were not yet observed in the treated flowers, although flowers treated with 50 mg L^-1^ NAg exhibited senescence on day 11 and flowers treated with 25 mg L^-1^ NAg showed senescence on day 13. Thus, 25 mg L^-1^ NAg was found to be the optimal concentration for extending vase life until day 12 (**Figure 2A**). Vase life was observed to be strongly linked to RFW as RFW of control flowers was lesser than RFW of treated flowers. Specifically, on day 3, RFW of control flowers was 94%, whereas flowers treated with NAg (25 and 50 mg L^-1^) had 106 and 101% RFW, respectively. On day 6, RFW of control flowers decreased by approximately 80% and the highest reduction (46.9%) was observed on day 9, whereas the treated flowers maintained their RFW until day 9 (**Figure 2B**). However, the RFW of 25 mg L^-1^ NAg treated flowers was higher than that of 50 mg L^-1^ NAg treated flowers throughout the vase period (**Figure 2B**).

### Bacterial Density, Identification of Bacteria, and SEM Observation

**FIGURE 3:**
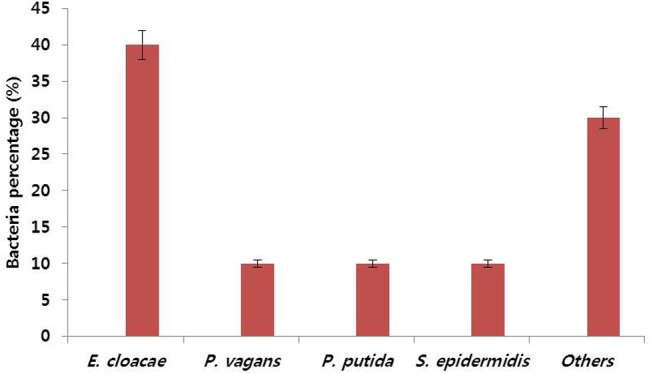
Percentages of populations of different types of bacteria observed in stem segments of control ‘Omea’ flowers. The data were taken on day 6 after treatment.

To verify the relationship between vase life and number of bacteria in the stem segments, bacterial numbers in the stem segments were determined on day 6, as most control flowers showed petal senescence or wilting on this day, which led to loss of ornamental value. We observed that bacterial density in the stems of control flowers was 2.7 × 10^10^ cfu mL^-1^ on day 6, whereas no bacterial colony was observed in the stems of NAg-treated flowers (**Table 2**). On the basis of the phenotypic characteristics of bacterial colonies, especially color and size of colonies, the bacteria were identified to be *E. cloacae* (40%), *Pantoea vagans* (10%), *Pseudomonas putida* (10%), *Staphylococcus epidermidis* (10%), and others (30%), by 16S rDNA-based PCR analysis (**Figure 3**).

**Table 2:** Comparison of bacterial concentrations inside stem segments of control and nano-silver (NAg) treated flowers.

Treatment	Bacterial concentration (cfu mL^-1^)
Control	2.7 × 10^10^
NAg (25 mg L**^-^**^1^)	0
NAg (50 mg L**^-^**^1^)	0


*Data were taken on day 6 after treatment (cfu mL**^-^**^*1*^ = colony forming unit per milliliter).*

**FIGURE 4:**
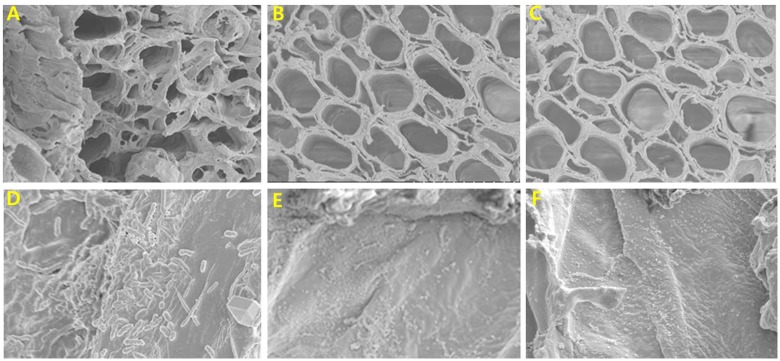
Comparison of cut-stem surfaces and xylem vessels of control **(A)**, 25 mg L^-1^ NAg treated **(B)**, and 50 mg L^-1^ NAg treated **(C)** flowers, and comparison of presence or absence of bacteria in walls of xylem vessels of control **(D)**, 25 mg L^-1^ NAg treated **(E)**, and 50 mg L^-1^ NAg treated **(F)** flowers. The photographs were taken on day 6 after treatment.

Scanning electron microscopy observations also revealed extensive bacterial colonization and biofilm formation on stem-end cut surfaces and in xylem vessels of the control on day 6, whereas treated flowers had a clear appearance (**Figure 4**). In addition, growth of different bacteria in the xylem vessel walls of control flowers was confirmed by SEM (**Figure 4D**), whereas almost no bacteria were observed in the xylem vessel walls of treated flowers (**Figures 4E,F**).

### Ethylene Production and Related Gene Expression

**FIGURE 5:**
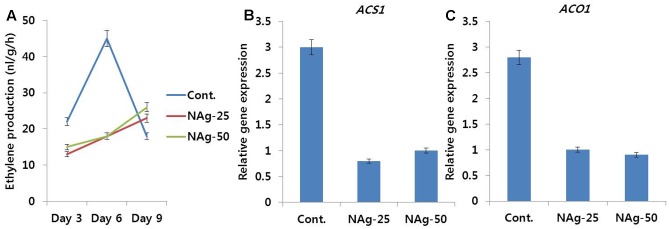
Effect of NAg on ethylene production **(A)** and transcript levels of 1-aminocyclopropane-1-carboxylate synthase (*DcACS1*) **(B)** and 1-aminocyclopropane-1-carboxylate oxidase (*DcACO1*) **(C)** genes in the petals of ‘Omea.’ Data for gene expression were collected on day 6 after treatment. Data represents the means ± SE of three replicates.

Even on day 3, ethylene production in control was higher than that in NAg treated flowers, and reached a peak on day 6 when most of the control flowers showed petal senescence, and afterward, rapid decline in ethylene production was observed on day 9 (**Figure 5A**), when the petal had completely senesced. In contrast to the control, the treated flowers produced low amounts of ethylene at the beginning of vase period and slightly increased the production rate by day 9; however, this increase in production rate was similar to the production rate detected on day 3 in the control. Most of the treated flowers survived until day 12, however, among all the treatments, lowest ethylene production was obtained on 25 mg L^-1^ NAg treatment. Analysis of transcript levels of ethylene biosynthesis genes (*DcACS1* and *DcACO1*) by qRT-PCR also validates the ethylene production rate observed in control and treated flowers (**Figures 5B,C**), as transcript levels of *DcACS1* and *DcACO1* in control petals were higher than the levels observed in treated petals. However, the transcript levels in 25 and 50 mg L^-1^ NAg treated flowers were not different.

### Expression Levels of CP1 and CPi Genes

**FIGURE 6:**
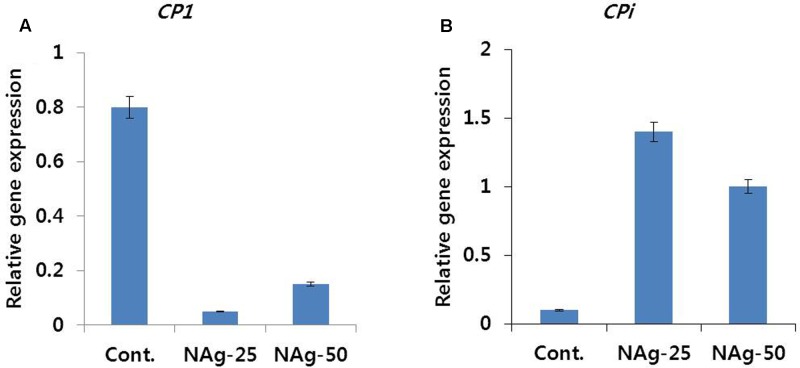
Effect of NAg on the transcript levels of cysteine proteinase (*DcCP1*) **(A)** and cysteine proteinase inhibitor (*DcCPi*) **(B)** genes in the petals of ‘Omea.’ Data were collected on day 6 after treatment. Data represents the means ± SE of three replicates.

Transcript levels of senescence-regulated (*DcCP1*) and senescence inhibitor (*DcCPi*) genes in control and treated flowers were different on day 6 (**Figure 6**). Expression level of *DcCPi* in treated flowers was higher than that in control (**Figure 6B**), whereas the expression level of *DcCP1* gene was associated with the degree of petal senescence (**Figure 6A**). In addition, the differences in transcript levels of 50 and 25 mg L^-1^ NAg treated flowers were also associated with the degree of petal senescence.

### Antioxidant Activities

**FIGURE 7:**
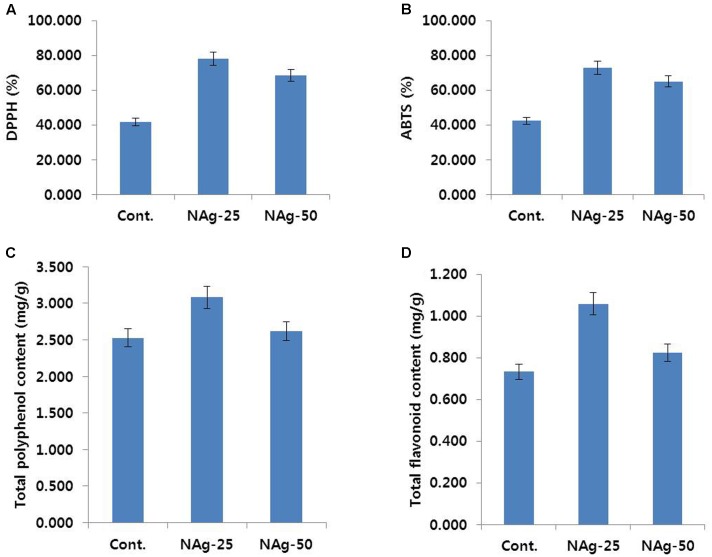
Effect of NAg on DPPH activity **(A)**, ABTS activity **(B)**, total polyphenol content **(C)**, and total flavonoid content **(D)** in the petals of ‘Omea.’ Data were collected on day 6 after treatment. Data represents the means ± SE of three replicates.

Generally, petal wilting or senescence is strongly associated with antioxidant activities, such as ROS-scavenging activities and total flavonoid and total polyphenol contents, in the petals. The control flowers exhibited petal senescence on day 6, whereas such senescence was not observed in NAg treated flowers on day 6. Thus, on day 6, we determined the ROS-scavenging activities using DPPH and ABTS assays, and analyzed the total polyphenol and total flavonoid contents in control and NAg treated flowers. As expected, the activities of DPPH and ABTS in the NAg treated flowers were higher than that in the control (**Figures 7A,B**). Specifically, the activities in 25 mg L^-1^ NAg treated flowers were the highest, followed by the activities in 50 mg L^-1^ NAg treated flowers, and then, in control flowers. Additionally, total polyphenol and flavonoid contents in both control and NAg treated flowers showed a trend similar to the trend exhibited by activities of DPPH and ABTS in these flowers (**Figures 7C,D**). From these findings, it is obvious that NAg treatments helped in maintenance of antioxidant activities leading to longer vase life, which resulted in delayed petal senescence of carnation.

### Effect of *E. cloacae* on Vase Life

#### Vase Life and RFW

**FIGURE 8:**
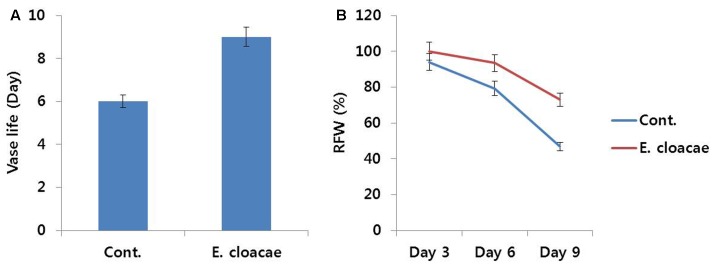
Effect of the bacterial strain *Enterobacter cloacae* on vase life **(A)** and RFW **(B)** of cut carnation flowers ‘Omea’ during the vase period. Data represents the means ± SE of three replicates.

According to the results of bacterial quantification and identification, percentage of *E. cloacae* in stem segments of control was the highest. Thus, we investigated the association of this bacterial strain with reduction in vase life. Addition of *E. cloacae* (10^7^ cfu mL^-1^) to 500 mL of the vase solution (distilled water) resulted in higher uptake of water by the stems as compared with control (data not shown). RFW of control slightly decreased from day 3 and a distinct decline was noticed on day 6. In contrast, addition of *E. cloacae* led to a significant increase in RFW until day 6, which decreased slightly thereafter (**Figure 8**). This trend mirrored the duration of vase life, as the vase life of treated flowers was extended by 3 days in comparison with the vase life of control (**Figure 8**). Moreover, the vase solution of treated flowers contained lesser *E. cloacae* and its pH value was also more acidic than that of control (data not shown).

#### Ethylene Production and Related Gene Expression

**FIGURE 9:**
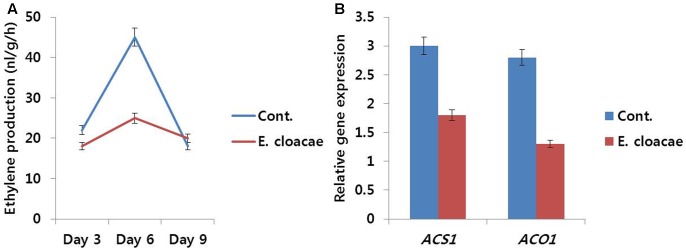
Effect of the bacterial strain *E. cloacae* on ethylene production **(A)** and the transcript levels of *DcACS1* and *DcACO1* **(B)** genes in the petals of ‘Omea.’ Data for gene expression were collected on day 6 after treatment. Data represents the means ± SE of three replicates.

Throughout the vase period, ethylene production in treated flowers was relatively lower than that in the control (**Figure 9A**), particularly on day 6, which was associated with the lower transcript levels of ethylene biosynthesis genes (*DcACS1* and *DcACO1*) observed in treated flowers as compared with the levels observed in control flowers (**Figure 9B**).

#### Expression of CPi and CP1 Genes

**FIGURE 10:**
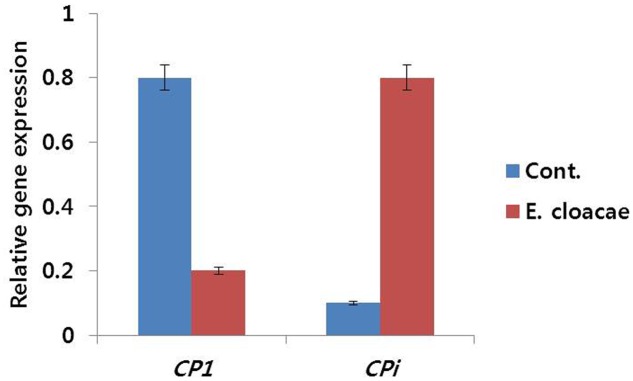
Effect of the bacterial strain *E. cloacae* on the transcript levels of *DcCP1* and *DcCPi* genes in the petals of ‘Omea.’ Data were collected on day 6 after treatment. Data represents the means ± SE of three replicates.

Expression levels of these genes were found to be associated with vase life of treated and control flowers. Senescence-regulated gene (*DcCP1*) showed high expression level in control, whereas, low expression level was observed in treated flowers. Conversely, the expression level of *DcCPi*, which inhibits early senescence, was lower in control than the level in treated flowers (**Figure 10**).

#### Antioxidant Activities

**FIGURE 11:**
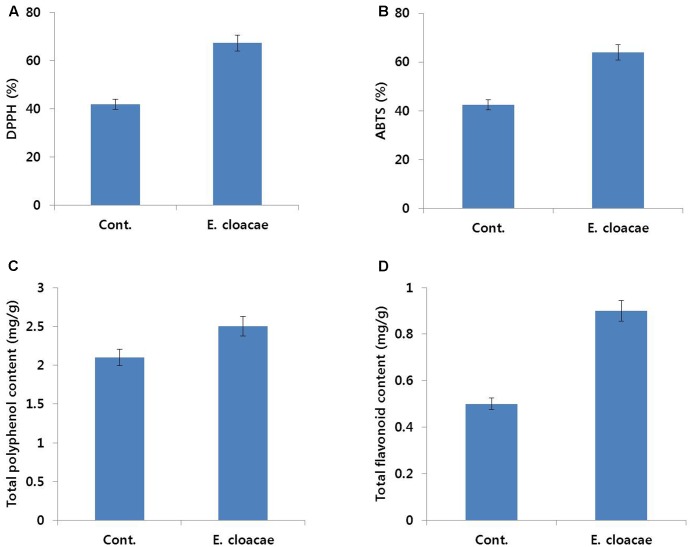
Effect of the bacterial strain *E. cloacae* on DPPH activity **(A)**, ABTS activity **(B)**, total polyphenol content **(C)**, and total flavonoid content **(D)** in the petals of ‘Omea.’ Data were collected on day 6 after treatment. Data represents the means ± SE of three replicates.

Results of antioxidant activity analysis also support the positive role of *E. cloacae* in extending vase life of carnation. Activities of DPPH and ABTS, which scavenge ROS, were higher in treated than control flowers. Similarly, total polyphenol and flavonoid contents were higher in treated than control flowers (**Figure 11**).

## Discussion

### Role of NAg in Extending Vase Life of Cut Flowers

In recent years, the application of NAg to cut flowers has greatly increased with evidence of its role in extending the vase life of various cut flowers, such as rose, gerbera, gladiolus, and carnation (Liu et al., 2009a,b; Li et al., 2012, 2017). NAg mainly acts as an antimicrobial agent that inhibits bacterial growth in xylem vessels of cut flowers, leading to increased water uptake by the cut stem, and consequently, increased vase life (Liu et al., 2009a; Li et al., 2012, 2017). However, most of the previous studies did not investigate the role of NAg in suppression of genes related to ethylene production, regulation of senescence-related genes, and maintenance of antioxidant activities, all of which are associated with vase life of cut flowers. In addition, positive effect of NAg on vase life of carnation has previously been reported by Liu et al. (2009b). However, the authors did not investigate the role of NAg in regulation of the above-mentioned factors. It would be interesting to investigate whether NAg participates in control of the biochemical and genetic mechanisms underlying the processes central to senescence of cut flowers. Therefore, in this study, we tried to understand the role of NAg in these mechanisms that determine vase life of cut carnation, and incorporated the missing links in the representative model of cut flower senescence.

In previous studies, optimal concentration of NAg required for flower longevity differed from crop to crop, as 25 mg L^-1^ NAg was found to be optimal for rose and 25 and 50 mg L^-1^ NAg were observed to be optimal for gladiolus (Li et al., 2012, 2017), whereas 5 mg L^-1^ NAg was found to be optimal for gerbera (Liu et al., 2009a). In our preliminary experiment, vase life of cut carnation varied with the different concentrations of NAg (1–50 mg L^-1^) used (data not shown), and 25 and 50 mg L^-1^ NAg were found to extend vase life of carnation as compared with control. Thus, we chose these two concentrations for analysis of the effect of NAg on vase life of cut flowers. In fact, in this study, 25 mg L^-1^ NAg was found to be better for increasing vase life and RFW than 50 mg L^-1^ NAg, which was not consistent with the results obtained by Liu et al. (2009b), who reported that 15 mg L^-1^ NAg exerts a positive effect on vase life of carnation. This discrepancy could be because of the use of different species or genotypes of carnation, or the examination of the effect of a single concentration of NAg (15 mg L^-1^) in their experiment. In addition, 50 mg L^-1^ NAg exerts a less positive effect on vase life and RFW than 25 mg L^-1^ NAg, which could be because of its toxic effect on the flower membrane; similar results were also reported in gerbera (Liu et al., 2009a). One of the main reasons for increase in vase life and RFW by NAg was its antibacterial activity, which suppresses bacterial growth in cut-stem surfaces and xylem vessels that transport water to flowers. This hypothesis is validated by the blockage of cut stem surfaces and xylem vessels due to high bacterial density (2.7 × 10^10^ cfu/mL) observed in control as compared with NAg treated flowers. A similar effect of NAg on bacterial growth in cut-stem surfaces and xylem vessels has been reported in rose, gerbera, and gladiolus (Liu et al., 2009a; Li et al., 2012, 2017). Isolation of bacteria from stem segments and its identification by 16S rDNA-based PCR analysis revealed that different types of bacteria [*E. cloacae* (40%), *Pantoea vagans* (10%), *Pseudomonas putida* (10%), *Staphylococcus epidermidis* (10%), and others (30%)], were mixed in the stem segments, and blockage by these different types of bacteria was confirmed by SEM. Among the isolated bacteria, *E. cloacae* is known to be a biocontrol agent that can kill the microorganisms in the vase water. However, it seemed that occurrence of high bacterial density (2.7 × 10^10^ cfu mL^-1^) in the stem segments led to blockage of the xylem vessels, which resulted in water deficit stress and petal wilting. Lethal wilting related to hampered water uptake, owing to xylem vessel blockage of the stem cut end, is aggravated in cut flowers with uncontrolled rates of water loss (Fanourakis et al., 2012). Decreased transpirational water loss by means of either very responsive stomata (Fanourakis et al., 2012) or employment of antitranspirant compounds (Fanourakis et al., 2016) has been shown to partly alleviate lethal wilting symptoms by maintaining a positive water balance for longer periods.

Many researchers have reported that NAg can extend the post-harvest life of various cut flowers (Liu et al., 2009a,b; Li et al., 2012, 2017). However, they did not determine the role of NAg in ethylene production, which plays an important role in senescence of cut flowers. In this study, NAg treatment significantly suppressed ethylene production as compared with the control, thus, another reason for increase in vase life by NAg could be its anti-ethylene activity. Higher amount of ethylene produced by 50 mg L^-1^ NAg treated flowers than by 25 mg L^-1^ NAg treated flowers could be because of its toxicity that resulted in higher ethylene production, and consequently, petal senescence. Reduction in ethylene production on NAg treatment has also been reported in lily and rose (Kim et al., 2005; Hassan et al., 2014).

Ethylene is produced via transcriptional activation of ethylene biosynthesis genes, such as *DcACS1* and *DcACO1* (Mostofi et al., 2010; Mortazavi et al., 2011; Liao et al., 2013). Kim et al. (2005) and Hassan et al. (2014) have reported that NAg suppressed ethylene production during the post-harvest life of cut lily and rose. However, they did not investigate the link between the rate of ethylene production and the related gene expression. In this study, we observed that NAg decreased ethylene production by suppression of transcript levels of *DcACS1* and *DcACO1*, as the transcript levels of these genes were observed to be the lowest in 25 mg L^-1^ NAg treated flowers, followed by 50 mg L^-1^ NAg treated flowers and control flowers. Recently, Tanase et al. (2013) reported that low ethylene production in carnation flowers was because of low transcript levels of *DcACS1* and *DcACO1*, and high transcript level of *DcACS1* and *DcACO1* was associated with an increase in ethylene production that was observed on the fifth and sixth day after treatment of carnation flowers (Tanase et al., 2008; Ichimura and Niki, 2014). Moreover, the difference in duration of vase life among different carnation cultivars was because of the difference in transcript levels of ethylene biosynthesis genes (Tanase et al., 2008).

Rate of ethylene production was directly associated with senescence of carnation flowers. Therefore, we assumed that the level of ethylene production could be related to the level of expression of senescence-related genes, such as cysteine proteinase gene (*DcCP1*), which leads to decomposition of cell components and cell death during petal senescence (Tanase et al., 2013), and cysteine proteinase inhibitor gene (*DcCPi*). As expected, in this study, high expression of *DcCPi* was observed in NAg treated flowers leading to low ethylene content. The expression of *DcCPi* was found to be highest in 25 mg L^-1^ NAg treated flowers, followed by 50 mg L^-1^ treated and control flowers. These levels corresponded to vase life of the cut flowers and supported the findings of Tanase et al. (2013, 2015), who reported that *DcCPi* acts as a suppressor of petal senescence in different carnation cultivars. In addition, Kim et al. (1999) and Sugawara et al. (2002) also demonstrated the role of *CPi* gene in senescence of carnation flowers. These results indicated that the regulation of senescence-related genes was associated with ethylene biosynthesis.

In this study, antioxidant activities, in terms of DPPH and ABTS activities, and total polyphenol and flavonoid content, were found to be higher in NAg treated flowers as compared with control flowers. Specifically, the highest activities could be detected in 25 mg L^-1^ NAg treated flowers. Higher antioxidant activity delayed petal senescence. It is likely that the antioxidants scavenged ROS induced by oxidative stress in cut flowers, which damages cell membranes. Comparatively, antioxidant activities were observed to be relatively lower in control flowers that exhibited earliest senescence (on day 6). These results are consistent with those of previous studies, which demonstrated that petal senescence is positively associated with the decline of antioxidant activities in flowers (Droillard and Paulin, 1987; Chakrabarty et al., 2007; Ezhilmathi et al., 2007; Kumar et al., 2008; Zeng et al., 2011; Dwivedi et al., 2016). This also supports the findings of Hassan et al. (2014), who reported that NAg could significantly extend the vase life of cut rose flowers by increasing antioxidant activities.

### Effect of *E. cloacae* on Vase Life of Carnation

In previous studies, petal senescence of cut flower was found to be directly or indirectly associated with presence of bacteria in vase solution or cut-stem surfaces (van Doorn et al., 1995; Liu et al., 2009a; Li et al., 2012, 2017). However, some studies have reported that addition of exogenous bacterial suspension (<10^8^ cfu mL^-1^) either had no effect or slightly reduced the longevity of cut carnation flowers (van Doorn et al., 1991, 1995), whereas Ratnayake et al. (2012) and Williamson and Joyce (2013) reported that high concentrations of bacteria did not influence vase life of cut *Boronia heterophylla* and *Acacia holosericea* flowers. These discrepancies in the findings obtained from the previous studies could be because of the different types of bacteria used in their experiments, since detailed studies on bacterial growth have revealed that shortening of vase life by bacteria depended on the type of bacteria present in the vase solution (van Doorn et al., 1991; Jacob and Kim, 2010; Carlson et al., 2015). In our study, a mixture of different types of bacteria was present on the cut-stem surfaces, out of which, percentage of *E. cloacae* was the highest (40%). However, the role played by the bacterial strain in determining vase life of cut flowers has not been well-documented in previous studies. Interestingly, addition of *E. cloacae* (10^7^ cfu mL^-1^) to the vase solution enhanced vase life of the carnation ‘Omea’ by 3 days in comparison with the control. Measurements of RFW and antioxidant activities also support the positive effect of *E. cloacae* on vase life. In addition, results obtained from analysis of ethylene production and related gene expression, as well as senescence-related gene expression, are also consistent with the positive effect of *E. cloacae* on vase life. van Doorn et al. (1995) reported that the concentration of bacteria (<10^8^ cfu mL^-1^) did not affect vase life of carnation, however, in this study, addition of 10^7^ cfu mL^-1^ bacteria enhanced vase life by 3 days as compared with the control. The differences between these two studies could be because of the use of different bacterial strains. Generally, *E. cloacae* is known to be an oxidase negative and catalase positive biocontrol agent of plant diseases. Thus, it is possible that *E. cloacae* enhances vase life by killing microorganisms present in the vase solution that can hasten flower senescence. In addition, another possible explanation is that its oxidase negative effect reduces ROS that are induced by oxidases, whereas its catalase positive effect enhances antioxidant activities leading to scavenging of ROS that damage plant cell membranes. Moreover, vase solution containing added *E. cloacae* was clearer in appearance than control vase solution, which could be because of the presence of fewer microorganisms and their decayed products. Recently, Carlson et al. (2015) also reported that two bacterial strains (*Pseudomonas fulva* and *Escherichia coli*), which are also known as biocontrol bacteria, enhanced vase life of cut *Zinnia elegans*. In this study, the possible reason for reduction of ethylene production would be explained that the bacteria may produce ACC deaminase to lower plant ethylene levels (Glick, 2014), and which may protect flowers against wilting, metals, organic contaminants, and bacterial and fungal pathogens (Glick, 2014). In addition, presence of fewer microorganisms and their decayed products in vase solution and xylem vessel by the bacteria would properly transport water to petals, and consequently would support preventing from premature wilting and high ethylene production.

Taken together, application of NAg that can suppress both ethylene and bacterial growth distinctly extended the vase life, In comparison with NAg, the bacterial strain less extended the vase life, which would be that as it could not directly suppress ethylene production as well as its ability to kill the microorganisms would not be as fast as nano-sliver.

## Conclusion

Nano-silver treatment enhanced longevity of the carnation ‘Omea’ by suppressing bacterial growth on cut-stem surfaces and in xylem vessels, reducing ethylene production and related gene expression, and maintaining antioxidant activities in comparison with the control. In addition, it suppressed the expression of *DcCP1* gene and enhanced the expression of *DcCPi* gene at transcriptional level, both of which are involved in petal senescence. 25 mg L^-1^ NAg treated flowers showed the best results. Moreover, it was observed that addition of the bacterium *E. cloacae* isolated from stem segments also enhanced longevity of carnation by giving better results than the control for all the analyzed parameters. This study demonstrates that NAg increases vase life of carnation through multiple modes of action. Moreover, *E. cloacae* played an important role in biocontrol of plant diseases and microorganisms causing petal senescence.

## Availability of Data and Materials

The datasets used and/or analyzed during the current study are available from the corresponding authors on reasonable request.

## Ethics Statement

*Carnation* is widely used as ornamental plant in plant biotechnology and horticultural research. This research was conducted in accordance with the regulations of the Korean Government.

## Author Contributions

AN designed the study, conducted the experiment, and wrote the manuscript. CK supervised experiments at all stages and performed critical revisions of the manuscript. KL, JH, and NW assisted with experimental procedures. All authors read and approved the final manuscript.

## Conflict of Interest Statement

The authors declare that the research was conducted in the absence of any commercial or financial relationships that could be construed as a potential conflict of interest.

## Abbreviations

ABTS: 2, 2′-azino-*bis*-3-ethylbenzthiazoline-6-sulfonic acid
ACO: 1-aminocyclopropane-1-carboxylate oxidase
ACS: 1-aminocyclopropane-1-carboxylate synthase
*CP1*: cysteine proteinase 1 gene
*CPi*: cysteine proteinase inhibitor gene
DPPH: 1, 1-diphenyl-2-picrylhydrazyl radical
1-MCP: 1-methylcyclopropene
NAg: nano-sliver
qRT-PCR: quantitative real time polymerase chain reaction
RFW: relative fresh weight
ROS: reactive oxygen species
STS: silver thiosulfate
